# Prediction of gene expression using histone modification patterns extracted by Particle Swarm Optimization

**DOI:** 10.1093/bioinformatics/btaf033

**Published:** 2025-01-29

**Authors:** Niels Benjamin Paul, Jonas Chanrithy Wolber, Malte Lennart Sahrhage, Tim Beißbarth, Martin Haubrock

**Affiliations:** Department of Medical Bioinformatics, University Medical Center Göttingen, Göttingen 37099, Germany; Clinic of Cardiology and Pneumology, University Medical Center Göttingen, Göttingen 37099, Germany; Joint Research Center for Computational Biomedicine, RWTH Aachen University, Aachen 52074, Germany; Department of Medical Bioinformatics, University Medical Center Göttingen, Göttingen 37099, Germany; Department of Medical Bioinformatics, University Medical Center Göttingen, Göttingen 37099, Germany; Department of Medical Bioinformatics, University Medical Center Göttingen, Göttingen 37099, Germany

## Abstract

**Motivation:**

Histone modifications play an important role in transcription regulation. Although the general importance of some histone modifications for transcription regulation has been previously established, the relevance of others and their interaction is subject to ongoing research. By training Machine Learning models to predict a gene’s expression and explaining their decision making process, we can get hints on how histone modifications affect transcription. In previous studies, trained models were either hardly explainable or the models were trained solely on the abundance of histone modifications. Based on other studies, which used histone modification patterns, rather than their abundance, to identify potential regulatory elements, we hypothesize the histone modification pattern in a gene’s promoter to be more predictive for gene expression. We used an optimization algorithm to extract predictive histone modification profiles.

**Results:**

Our algorithm called PatternChrome achieved an average area under curve (AUC) score of 0.9029 over 56 samples for binary classification, outperforming all previous algorithms for the same task. We explained the models decisions to deduce the effect of specific features, certain histone modifications or promoter positions on transcription regulation. Although the predictive histone modification patterns were extracted for each sample separately, they can be used to predict gene expression in other samples, implying that the created patterns are largely generalizable. Interestingly, the impact of histone modifications on gene regulation appears predominantly indifferent to cellular specificity. Through explanation of the classifier’s decisions, we substantiate established literature knowledge while concurrently revealing novel insights into the intricate landscape of transcriptional regulation via histone modification.

**Availability and implementation:**

The code for the PatternChrome algorithm, the scripts for the analyses and the required data can be found at (https://gitlab.gwdg.de/MedBioinf/generegulation/patternchrome).

## Graphical abstract

**Graphical abstract:**
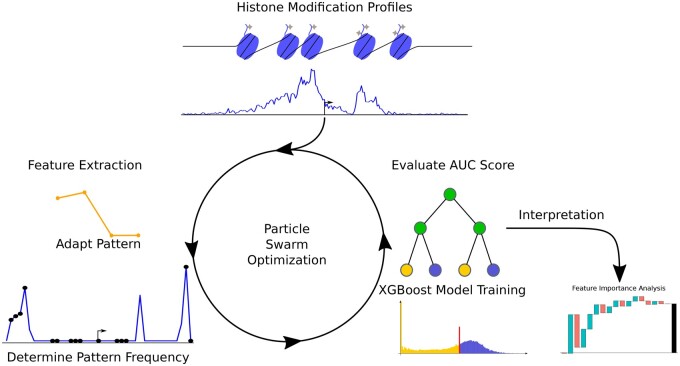

## 1 Introduction 

Histone positioning and their posttranslational modifications play a pivotal role not only in the organization of the DNA but also in the activation or repression of transcription. In the past, correlation analysis has been used to investigate the effect of specific histone modifications on transcription Barski *et al.* (2007). These studies revealed general trends for certain histone modifications like H3K4me3 which is overrepresented in the promoter regions of transcribed genes or the repressive histone modification H3K27me3 which marks heterochromatin Bannister and Kouzarides (2011). But transcription regulation by histone modifications is complex due to multiple factors: various modifications, including methylation, acetylation, ubiquitination, etc., can occur at different positions on histones. These modifications are not placed independently from each other, e.g. H3K4me3 is often placed in proximity to histone acetylation marks Zhang *et al.* (2015). Furthermore, the effect of a certain modification can be ambiguous. In order to study the complex effect of histone modifications, researchers started to train machine learning models with the aim of predicting gene expression from a set of histone modification data.

Cheng *et al.* used a Support Vector Machine to classify whether genes in *C. elegans* were expressed more than the median gene expression based on histone modification and DNA-binding protein ChIP-seq data and found that histone data adjacent to the transcription start site (TSS) and the transcription termination site were most predictive of gene expression Cheng *et al.* (2011).

A random forest classifier was used instead of a median cutoff by Dong *et al.* to classify genes as “on” or “off” based on data from chromatin assays Dong *et al.* (2012). Additionally, the authors used the “bestbin” approach together with a linear regression model to further quantify the “on” genes. Interestingly, histone modifications were of varying importance in these two steps.

Recent advances in the field of deep learning led to the development of DeepChrome by Singh *et al.*, a model using a deep convolutional neural network to classify gene expression based on histone modification data Singh *et al.* (2016). DeepChrome was trained on each of the 56 samples taken from the REMC (Roadmap Epigenomics) database Bernstein *et al.* (2010). As data input the histone signals of five histone modifications 5000 bp up- and downstream of the TSS were divided into bins with a length of 100 bp. DeepChrome outperformed previous studies in a direct comparison and was able to determine the regions and combinatorial histone modification patterns predictive of gene expression Singh *et al.* (2016).

In 2022, Frasca *et al.* developed the model ShallowChrome, that surpassed the prediction performances of previous prediction models. As indicated in the model name, the authors focused on the feature extraction part and used a less complex classification pipeline as opposed to prior deep learning models. Next to a superior performance, the model was also more explainable than its predecessors Frasca *et al.* (2022).

Current algorithms offer limited possibilities to determine the importance of specific promoter sub regions in regulating transcription. Therefore, we developed an interpretable method that utilizes patterns in histone mark ChIP-seq profiles to predict whether a gene has low or high gene expression levels. Since nucleosomes prevent most transcription factors from binding occupied DNA-motives we hypothesize that the histone positioning is more relevant for transcription activation than the amount of histone modification ChIP-seq signals. In order to use features that represent nucleosome positioning, we looked for scale-invariant predictive patterns in histone ChIP-seq data rather than the scale-variant amounts. The hypothesis that patterns in ChIP-seq data possess predictive power for gene expression is also supported by the fact that patterns in chromatin sequencing data have already been used to identify regulatory relevant regions Sethi *et al.* (2020). Since the search for patterns flexible in shape, width, and histone modification type required a huge feature space we used an optimization algorithm to extract the predictive features for our classifier. We chose Particle Swarm Optimization (PSO) as optimization algorithm with the objective of maximizing the predictive performance of our classifier. The extracted patterns serve as input features for an XGBoost classifier whose tree like nature together with the low level of abstraction, compared to a neural network, of the features enabled us to investigate the association between histone modifications and transcription. After comparing the performance of this approach to two of the most recent and best performing predecessor algorithms we made use of the XGBoostExplainer package (https://github.com/AppliedDataSciencePartners/xgboostExplainer) to investigate on the one hand the histone modification’s influence on the classifier and on the other hand at which promoter sub regions the occurrence of certain HM patterns is most decisive for transcription.

## 2 Materials and methods

### 2.1 Data collection and preprocessing

In our study a similar setup, as in Singh *et al.*, was used Singh *et al.* (2016). The processed ChIP-seq data from five histone modification and the gene expression data of 56 samples were downloaded from the REMC database Bernstein *et al.* (2010). The samples are a mixture of primary cell lines, primary cultured cell lines, and embryonic stem cell-derived cell lines. Meta information provided by REMC is available on this website: (https://docs.google.com/spreadsheets/d/1yikGx4MsO9Ei36b64yOy9Vb6oPC5IBGlFbYEt-N6gOM/edit#gid=15). All data were already passed through multiple preprocessing steps via REMC to guarantee good quality and standardized output. The ChIP-seq reads were mapped to the hg19 reference genome and were subsequently truncated to 36 bp. These 56 samples were selected because the histone modification data for the five chosen histone modifications were available for these samples. The five histone modifications were chosen as they were used in previous studies to allow a direct and fair comparison. The five selected histone modifications are shown in Table 1.

**Table 1.** Characteristics of the five investigated histone modifications.

Histone modification	Associated with	Major effects
H3K4me3	Promoter regions	Promote gene transcription Wysocka *et al.* (2006)
H3K4me1	Promoter/Enhancer regions	Enhancer fine-tuning, gene expression maintenance Rada-Iglesias (2018)
H3K36me3	Gene-transcribed regions	DNA repair Pfister *et al.* (2014), gene expression stability Pu *et al.* (2015)
H3K9me3	Heterochromatin regions	Transcriptional repression via heterochromatin formation Becker *et al.* (2016)
H3K27me3	Heterochromatin regions	Transcriptional repression via heterochromatin formation Francis *et al.* (2004)

The gene expression data were quantified from RNA-seq reads using the reads per kilobase of exon model per million mapped reads (RPKM) measure Mortazavi *et al.* (2008). Details about the REMC preprocessing steps are described on the REMC website (https://egg2.wustl.edu/roadmap/web_portal/processed_data.html).

### 2.2 Methods

#### 2.2.1 Data preprocessing

The aim of the algorithm is to classify genes according to their gene expression, in a binary prediction task. For this, the median gene expression for each sample was taken as a threshold and genes with a gene expression level above this threshold were indicated as genes with high gene expression and *vice versa*.

The gene expression data were provided for 19 795 genes for each of the 56 samples. The genes were referenced by their GENCODE identifier Frankish *et al.* (2021). Gene coordinates from RefSeq were used for mapping the histone signal reads relative to the TSS O’Leary *et al.* (2016). In order to associate the histone signal data with the gene expression data, the RefSeq gene annotations were converted into GENCODE identifiers. 18 421 genes could be mapped between GENCODE and RefSeq, while 1374 genes were lost with no GENCODE equivalent. The study used all 18 421 for which the histone signal data could be assigned to the proper gene expression data. We defined the prediction of gene expression similar to the setup by Singh *et al.* (2016). We binarized the gene expression data of each cell line taking the median gene expression level as threshold and categorizing the lower half of genes as lowly expressed and categorizing the upper half of genes as highly expressed. Prior authors Frasca *et al.* (2022) noted that taking the median as a cutoff may not be the biologically most relevant cutoff and, therefore, we also implement our approach as a regression task and using an alternative, biologically likely more relevant cutoff in the Supplementary Data S1, available as supplementary data at *Bioinformatics* online.

**Figure 1.**
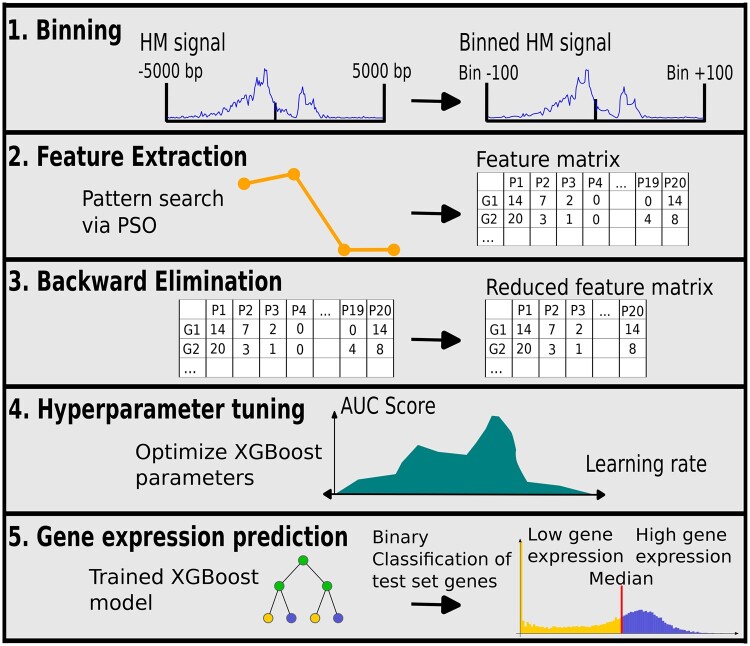
PatternChrome workflow. Histone modification data were restricted to the region around the TSS and were binned by summarizing 50 bps by calculating their mean. During feature extraction, PSO was used to find histone modification patterns predictive of gene expression in the training set. Next, in the BE step, redundant patterns were removed. In the Hyperparameter tuning step PSO was employed again to find optimal parameters for the XGBoost classifier. In the final gene expression prediction, the final XGBoost model predicted the gene expression in the test set.

Similar to Singh *et al.* (2016), the region 5000 bp up- and downstream of the TSS of each gene was selected and divided into bins. In contrast to Singh *et al.*, who used bins of a length of 100 bps, a length of 50 bps was chosen in this study to capture more distinct patterns. A bin represents the average histone signal across this 50 bps. Thus, 200 bins for each of the five histone modifications were used as input features for the classification model. An example of the input data for one gene is shown in Fig. 1.

Genes were divided into training, validation and test set. In order to be comparable to DeepChrome, the training set contained 6600 genes, while the rest of the genes were equally split into validation (5911 genes) and test set (5910 genes). Although DeepChrome used 6601 genes for training, we decided to use 6600 genes, since only an even number of training genes enabled balanced labels during training. The genes for each set were randomly sampled for each sample separately to ensure balanced labels in the test set.

#### 2.2.2 General approach

The classification process of PatternChrome can be divided into four steps: (i) Feature extraction, (ii) Backward elimination (BE), (iii) Hyperparameter tuning, (iv) Final XGBoost classification. A general workflow is shown in Fig. 1. The following sections describe each step in more detail. The code was implemented in R version 4.3.1 and run on a SMP Debian 5.10.209–2 (2024–01-31) x86_64 GNU/Linux system with the 5.10.0–28-amd64 kernel. As mentioned in the availability and implementation statement in the abstract, the implementation of these steps is available via GitLab. XGBoost Chen *et al.* (2024) was implemented with the R package xgboost (https://CRAN.R-project.org/package=xgboost). PSO was implemented using the R package pso Bendtsen (2022). The standard version of PSO from the year 2007 (SPSO2007) as described by Clerc (2012) was used.

#### 2.2.3 Feature extraction

**Figure 2.**
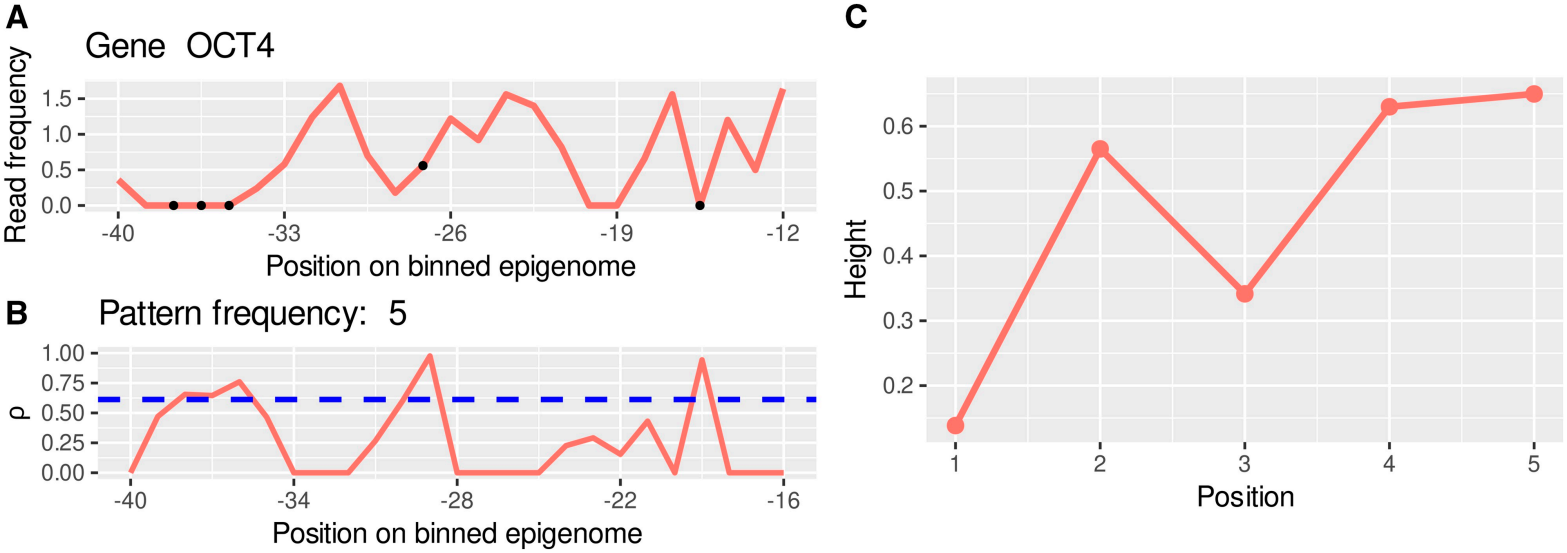
Visualization of the correlation between binned histone read signals and a pattern used for gene expression classification in sample E003. (A) Displays the summarized H3K4me3 ChIP-seq read profile for the region spanning 40–12 bins (1 bin = 50 bp) upstream of the OCT4 TSS in sample E003. The dots in A indicate matches between the ChIP-seq profile and a pattern used for feature extraction. This pattern, selected as an example, covers 5 bins (*x*-axis) and is characterized by values ranging from 0 to 1 at each point. (B) Depicts the Pearson correlation coefficient for the correlation between each position in the histone modification profile (A) and the subsequent 4 bins, as well as the pattern (C). The dashed line indicates the correlation coefficient cut-off used for this particular pattern. This cut-off is exceeded in 5 positions, and this number is counted as pattern frequency, contributing as an input feature for the subsequent classification task.

During feature extraction histone modification patterns predictive of gene expression are searched and their frequency in different genes is stored. The feature extraction of PatternChrome combines an optimization procedure, namely PSO, with a classification algorithm, namely XGBoost. Each feature extraction round we optimize for up to 11 parameters (see Table S3, available as supplementary data at *Bioinformatics* online for all the parameters) which are used to find a histone modification pattern whose frequency in the different genes in the training set can best be used to classify genes into above and below the median gene expression level using the XGBoost model. One pattern may occur on average more often in genes with above average gene expression and a higher pattern frequency for a gene may cause the XGBoost model to classify the gene as having an above median gene expression level. The abundance of a pattern in a histone modification profile is evaluated using Pearson’s correlation coefficient. For example, in Fig. 2 the pattern in the right subfigure can be found on five distinct positions in the selected histone modification profile given a correlation coefficient threshold of 0.6, i.e. there are five positions in the histone signal data whose correlation coefficient is higher than 0.6 when the histone signal data at the position is correlated with the pattern. The abundance of a pattern around the TSS of each gene is added to the abundance of patterns extracted in possible previous iterations and together the vector of pattern abundances is used as an input to train a XGBoost model on a random subset of the training gene set.

**Figure 3.**
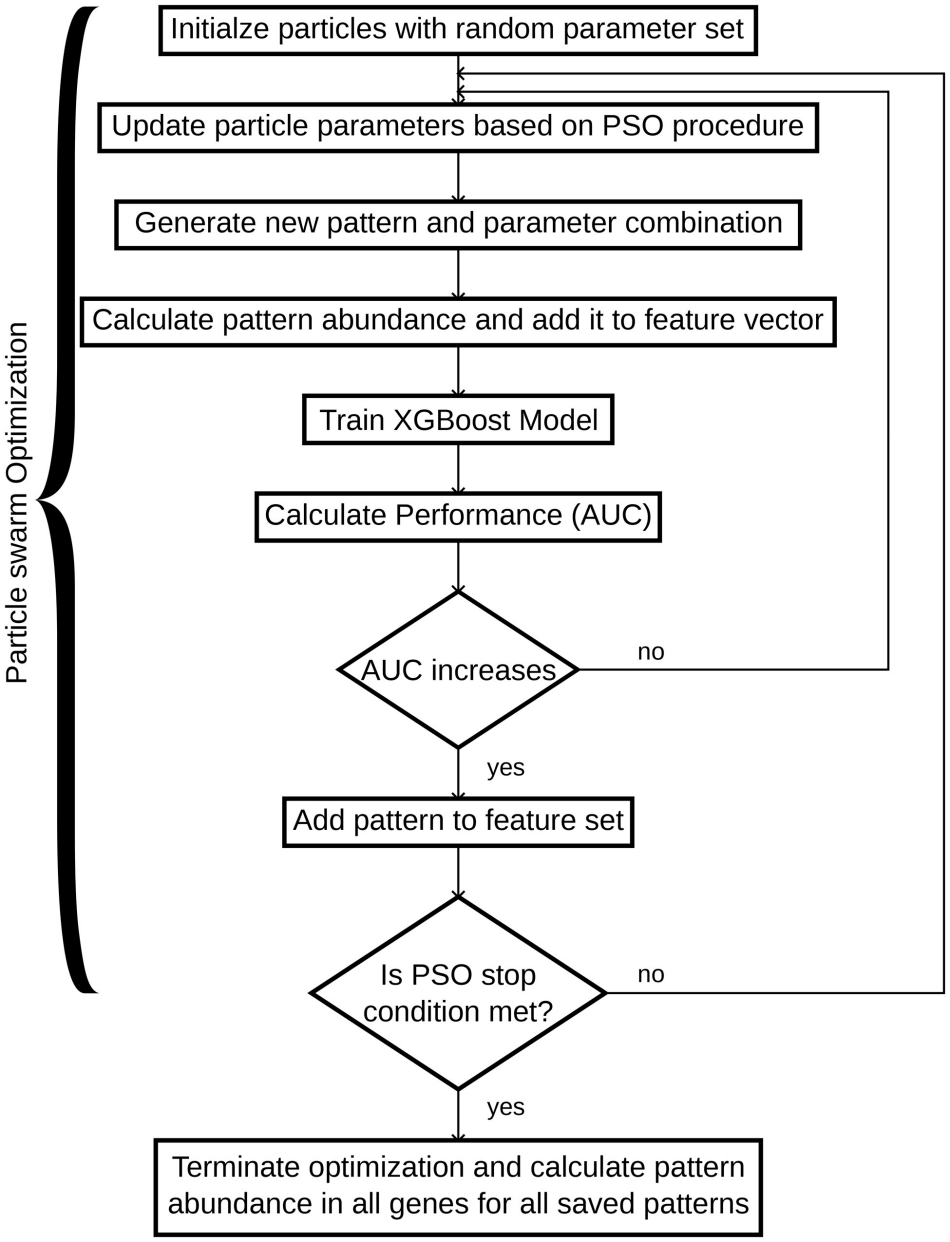
Flow diagram illustrating the PSO based feature extraction workflow.

During one PSO round several patterns are evaluated and at the end only the pattern is added that leads to the highest increase in the area under curve (AUC) score. The models performance is evaluated on a random and changing subset of 3000 genes of the training gene set. The XGBoost classifier runs for 50 iterations and the step size shrinkage parameter is set to 0.2. All other parameters are set to the default parameters of the XGBoost model. The PSO algorithm is stopped, when the AUC score on the training data exceeds a specified performance or a maximum number of iterations is reached without any improvement in the AUC score. Figure 3 shows an overview of the feature extraction workflow. Additional details about the PSO optimization process can be found in the section PSO implementation details in the Section S9.1, available as supplementary data at *Bioinformatics* online.

#### 2.2.4 Backward elimination

After feature extraction, BE is performed to remove redundant patterns from the classifier input. This is done by repeatedly training an XGBoost model on the feature matrix (i.e. the frequency of the optimized patterns) of the training set using the same XGBoost settings as in the feature extraction step. The model is then used to predict gene expression levels of the validation set. At the beginning of BE, all patterns are considered and an AUC score is calculated from all features (i.e. all patterns are included). Subsequently, the pattern last added to the feature matrix is transiently removed and a new AUC score is calculated. When the new AUC score is equal or greater than the previous AUC score the pattern is permanently removed. When the AUC score is worse than the previous AUC score, the pattern is added back to the feature matrix. The best AUC score is memorized and serves as a cutoff for further decisions. BE continues to temporarily remove patterns beginning from the last and ending with the first pattern. After a pattern is permanently removed BE starts again from the last pattern in the feature matrix. BE is finished when the algorithm has moved through all patterns without removing any.

#### 2.2.5 Hyperparameter tuning

During hyperparameter tuning, the hyperparameters of the XGBoost classification algorithm are optimized on the validation set. Hyperparameter tuning is performed via PSO and the optimization algorithm uses the same PSO parameter settings as in the feature extraction process.

#### 2.2.6 Final gene expression prediction using XGBoost classification

In the last step, the performance of the classifier for a particular sample is evaluated on the test set using the XGBoost classification algorithm. Just as in Singh *et al.* (2016) the AUC score was used as evaluation metric. For the prediction, an XGBoost model is trained on the training set using the parameter values provided by hyperparameter tuning and the features that passed BE. Subsequently, the resulting XGBoost model is used to predict the gene expression level of the test set genes.

#### 2.2.7 Performance comparison

The Performance, measured as AUC, was compared using pairwise Wilcoxon signed rank tests with continuity correction with a directional hypothesis implemented as *wilcoxon.test()* function Bauer (1972); Hollander *et al.* (2013) in the R package *stats*  R Core Team (2024).

## 3 Results

### 3.1 Performance evaluation

**Figure 4.**
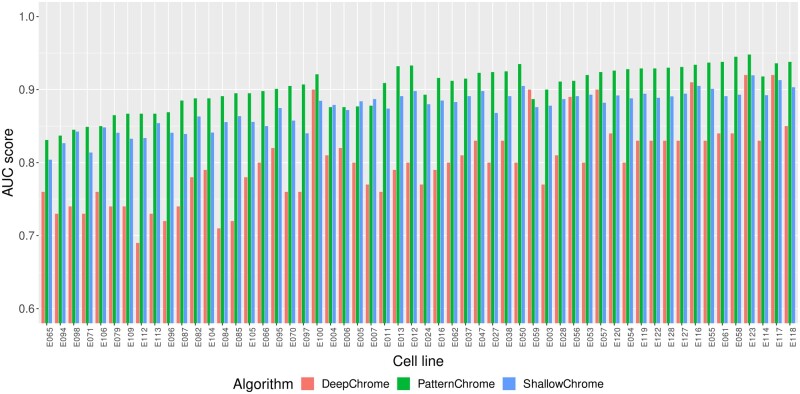
AUC scores of PatternChrome and two predecessing algorithms DeepChrome and ShallowChrome for all 56 samples. PatternChrome outperforms the predeceasing algorithms in 52 out of 56 samples with respect to AUC.

The results of the PatternChrome algorithm are compared to two of its predecessor algorithms: DeepChrome Singh *et al.* (2016) and ShallowChrome Frasca *et al.* (2022), which aimed to solve the same task of predicting the gene expression based on the same five HMs in the same dataset. PatternChrome performed consistently better than any of the predecessor algorithms (DeepChrome-PatternChrome: $p=4.08\cdot 10^{-11}$, ShallowChrome-PatternChrome: $p=8.23\cdot 10^{-11}$). It achieved the greatest AUC score in 52 out of 56 samples among the three compared algorithms. Figure 4 visualizes the differences in AUC score between the three algorithms. PatternChrome achieves the highest test AUC score in cell line E123 (AUC score = 0.9479) while it performs worst for cell line E065 (AUC score = 0.8313).

Table 2 displays the descriptive statistics of the AUC performances of the three compared algorithms. PatternChrome achieves the highest mean AUC score (0.0292 gap), the highest median AUC score (0.0271 gap), the best maximal performance (0.0254 gap) and the best minimal performance (0.0229 gap). The ShallowChrome algorithm is the most consistent algorithm among all three as indicated by the lowest AUC score standard deviation (0.0035 gap). Classification metrics for every individual cell line are given in Table S6, available as supplementary data at *Bioinformatics* online.

**Table 2.** Aggregated descriptive statistics of the AUC all three different prediction algorithms.

	DeepChrome	ShallowChrome	PatternChrome
Mean	0.8008	0.8737	**0.9029**
Median	0.8009	0.8829	**0.9100**
Max	0.9225	0.9196	**0.9479**
Min	0.6854	0.8084	**0.8313**
SD^a^	0.0553	**0.0260**	0.0295

a Standard deviation.

For each statistic, the value with the best result is marked in bold.

### 3.2 Analysis of feature importance

**Figure 5.**
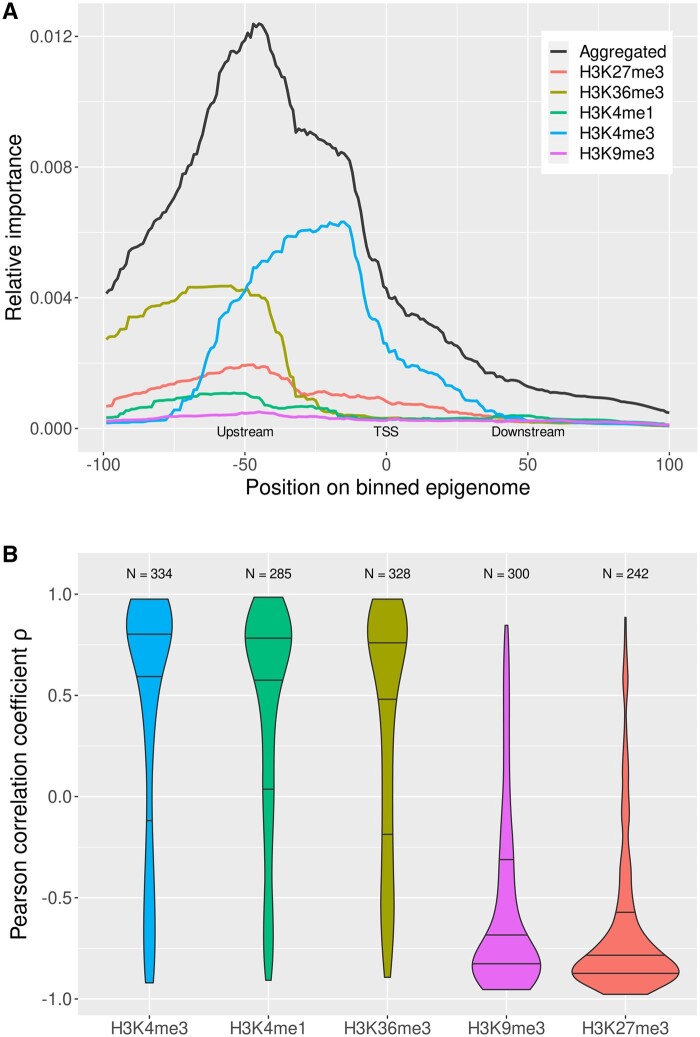
Feature importance and feature correlation analysis. (A) Relative importance of epigenomic regions for each individual histone modification and aggregated. The graph shows that the region ∼−50 bins up to the TSS is most important for the XGBoost model. (B) Feature correlations for individual histone modifications. The feature correlation is a measure of how the feature frequency is associated with the prediction. For H3K4me3 H3K36me3 and H3K4me1, most features are positively correlated indicating that these histone modifications may generally have a stimulating effect on gene expression whereas H3K9me3 and H3K27me3 may more often have an inhibitory effect on gene expression.

Using xgboostExplainer (https://github.com/AppliedDataSciencePartners/xgboostExplainer) package, we can break down the feature contributions to a single prediction, offering a level of explainability comparable to a single decision tree. The xgboostExplainer package offers insights into the log-odds contributions of each feature for each prediction. Log-odds contributions allow us to see exactly how each feature affects the likelihood of a gene being classified as expressed. Unlike the general feature importance for the XGBoost model where each feature importance is the average absolute contribution for all of the samples, the xgboostExplainer package breaks down the feature contributions for a decision (each individual gene prediction). The individual feature contribution can tell whether a feature has a positive or a negative impact on gene expression. We calculated the “relative importance” of each bin by summing up the feature importance from all models and dividing the aggregated importance at each position by the total number of bins. This final “relative importance” value thus represents the average contribution of each bin to all the model’s predictions, standardized across the entire TSS region, and allows for a straightforward comparison of influence across regions. Detailed information how the relative importance of each bin was computed is given in the Supplementary Data S1 in the section “Calculation of realtive feature importance values”, available as supplementary data at *Bioinformatics* online. Figure 5A visualizes the relative importance of each of the 200 bins representing the epigenome region for the five histone modifications. The bins most important to the XGBoost classification model are bins −64 to −32 representing the regions 3200–1600 bp upstream of the TSS. Although this region covers only 16.5% of the whole input region it has a cumulative importance to the classifier of 36.68%. The bin with the highest amount of aggregated feature importance’s is bin −45 representing the region 2800–2750 bp upstream of the TSS. For the XGBoost classifier the input bins upstream of the TSS has an aggregated importance of 82.95% while the regions downstream have an aggregated importance of 17.05%.

Figure 5 shows that the combined importance of all histone modifications aligns closely to the sum of the importance of the histone modifications H3K4me3 and H3K36me3. H3K4me3 is the most significant factor in determining importance around the TSS and up to 50 bins (−2500 bps) upstream. Upstream of this region, the importance shifts towards the histone mark H3K36me3. H3K27me3 and H3K4me1 have their greatest contributions around the −50 bin (2500 bps) upstream of the TSS. The histone modification H3K9me3 has generally a low importance and does not seem to be considerably enriched in a particular region.

To investigate the net effect of patterns grouped by their occurrence in different histone modifications, the distribution of the correlations between pattern frequency and XGBoost feature contributions grouped by the histone modification is plotted in Fig. 5. The histone modification H3K4me3 has the greatest net positive effect with an average *ρ* of 0.4183 ± 0.6151, H3K36me3 (*ρ *= 0.3519 ± 0.5801) and H3K4me1 (*ρ *= 0.4164 ± 0.5439) have similar effects as H3K4me3. Net positive effect means that with an increase in patterns for these histone modifications the classifier tends to label a gene as above median expressed. On the other hand, H3K27me3 and H3K9me3 have net negative effects with correlations of −0.6483 ± 0.3840 and −0.5530 ± 0.4483, respectively. All distributions have a high standard deviation indicating that patterns occurring in a histone modification can be both activating or repressive for gene expression.

### 3.3 Feature contributions for individual genes

In order to understand how the XGBoost model decides whether a gene has a high or a low gene expression level the feature contributions for an individual gene are determined. To demonstrate how feature contribution differ between active and inactive genes, one gene with a high and one with a low gene expression level were selected as an example.

**Figure 6.**
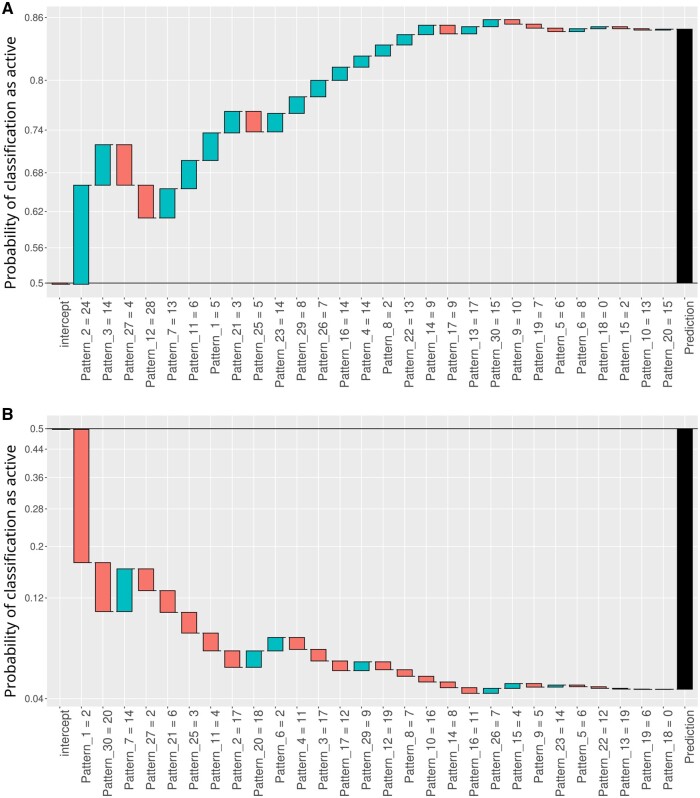
Waterfall plot displaying the contributions of the individual features in the XGBoost model prediction for individual genes. The contributions were calculated using the xgboostExplainer package. The *x*-axes display the amount of detected patterns in the ChIP-seq data sorted by importance (absolute of contribution). These values serve as features for the classification task. The *y*-axis represents the cumulative log-odds contributions from each feature, and summing these contributions yields the prediction probability (the threshold of 0.5 indicates the decision threshold between classification into genes expressed above or below median gene expression). (A) Waterfall plot for the OCT4 gene. The XGBoost model will predict a high gene expression level for the OCT4 gene as the sum of all individual feature contributions is above 0.5. (B) Waterfall plot for the TIE1 gene. In contrast to OCT4, the XGBoost model will predict a low gene expression level, as the sum of all feature contributions is below 0.5.

Just as in the previous section, cell line E003, an embryonic stem cell line, is used to train the model and predict gene expression. Oct4 is known to be highly expressed in stem cell lines as it has the function to maintain (and regain) pluripotency Shi and Jin (2010). And as expected, in cell line E003 Oct4 has a high gene expression level. Figure 6A shows the contributions of the individual patterns for the XGBoost model when predicting the gene Oct4. The XGBoost model succeeds in correctly predicting the high gene expression level as seen from the fact that the probability estimated by the model that this gene belongs to the group expressed above median is greater than 0.5. The model starts off with a negative intercept (value = −0.0085) which is the same for all genes. The individual pattern contributions are ordered by their importance. In the case of Oct4, Pattern 2 is the most important pattern which occurs 24 times in the investigated epigenome of Oct4. The fact that Pattern 2 occurs 24 times leads to a positive contribution of 0.6775. The final prediction value is calculated by the summing up the individual pattern contributions. As a counterexample, the breakdown of the classification of the TIE1 gene is shown in Fig. 6B. TIE1 encodes an tyrosine-protein kinase receptor involved in the regulation of angiogenesis and is expressed mainly in endothelial cells and hematopoietic stem cells Puri and Bernstein (2003). As a result, Tie1 is characterized by low gene expression in the E003 embryonic stem cell line. Pattern 1 was found twice for the TIE1 gene in this sample and had the largest contribution for the correct classification. While pattern 1 was found 5 times for the OCT4 gene and had a positive contribution, the fact that it was found just 2 times for the TIE1 gene led to a strong negative contribution. The same trend is observed for the most important pattern in the OCT4 classification, pattern 2. While it was found 24 times in the OCT4 gene and had a strong positive effect, the fact that it was found just 17 times in the TIE1 gene led to a slight negative contribution.

### 3.4 Pattern generalizability

**Figure 7.**
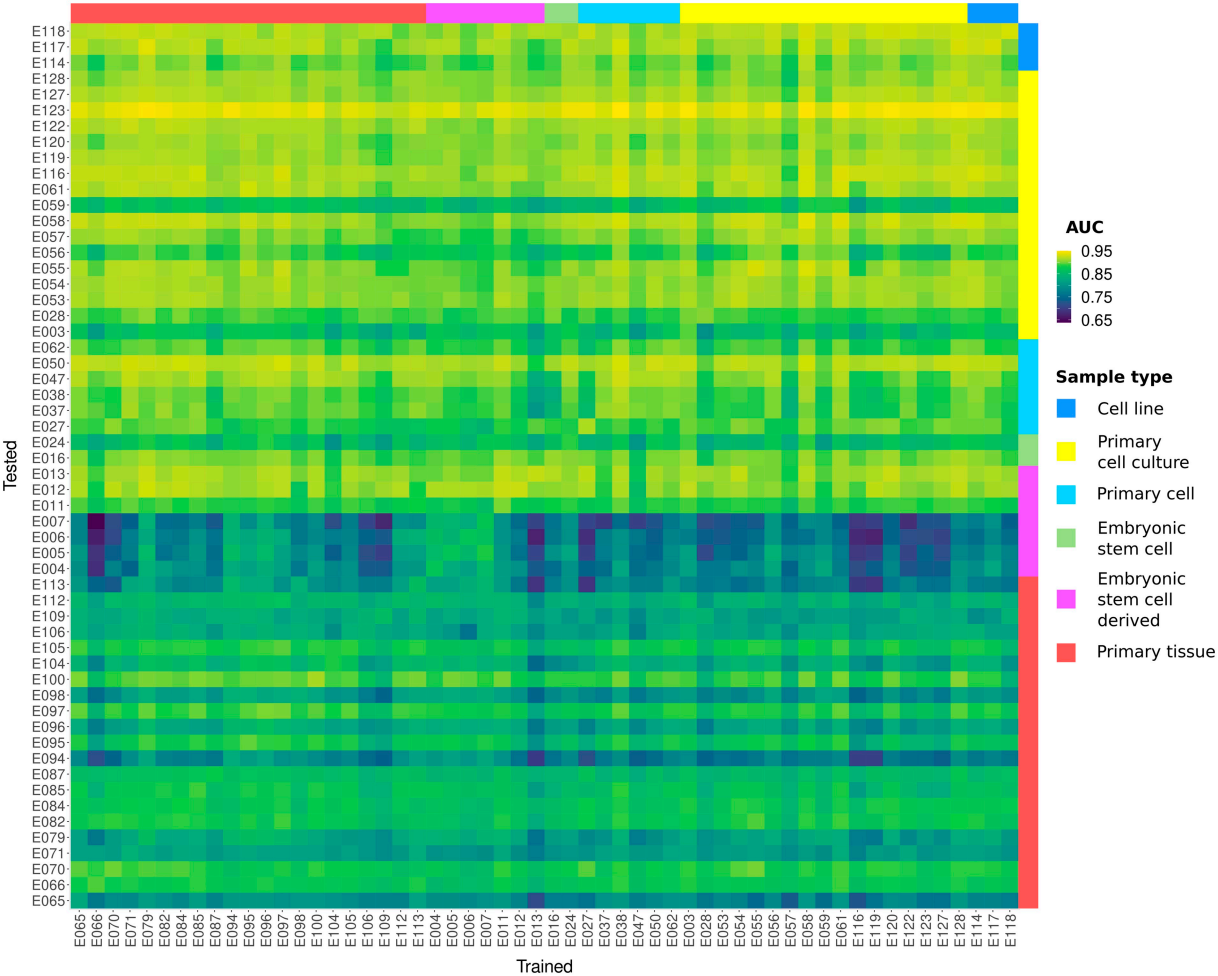
Heatmap displaying the AUC scores for each sample combination. For the individual AUC scores, the XGBoost model was trained on the training set of the genes on the vertical axis and predicted the test set of genes on the horizontal axis. Samples are grouped by sample type. The colors above and on the right side of the heatmap indicate the sample type of the samples. The heatmap demonstrates that the achieved AUC score mostly depends on the sample to which the model is applied and not on the sample it was trained on. Furthermore, the predictablilty of the samples differs between the sample types.

A key question is whether the learned patterns are specific for each sample or whether they can be generalized to predict gene expression across different samples. To investigate the generalizability of the patterns, the optimized features and the learned model of one sample were used to predict the gene expression in the test sets of all other 55 samples without any additional hyperparameter tuning or BE. Repeating this procedure for all 56 samples yielded 56 · 56 (= 3136) AUC scores. Figure 7 displays all these AUC scores in a heatmap. The average AUC score for when trained and tested sample are the same is 0.9029 ± 0.0297. In contrast, the average AUC score for when trained and tested cell lines are different is 0.8740 ± 0.0505. Although this AUC score is considerably lower, the AUC score is high enough to come to the conclusion that the learned patterns are very generic and only a small proportion of the learned patterns may be sample specific.

The distinctive blue vertical stripes in Fig. 7 also indicate that there are some cell lines that are generally hard to predict such as E007 (mean AUC score 0.7656 ± 0.0492). On the other hand, there are also cell lines which show consistently high AUC scores such as E123 (mean AUC score 0.9422 ± 0.0086). A possible explanation for these differences in sample predictability may be the sample type and the associated differences in cell homogeneity of the different samples (see the Section Tissue Type S9.3.1, available as supplementary data at *Bioinformatics* online for a more detailed analysis).

## 4 Discussion

This study aimed to develop and present a model capable of predicting gene expression from epigenetic data while also providing insights into the reasoning behind its predictions. PatternChrome uses an innovative feature extraction step via the use of PSO to extract those features from the epigenetic input data that are most predictive of gene expression. It then proceeds to classify the genes using the XGBoost algorithm. PatternChrome outperforms previous prediction algorithms using the same setup. Over all 56 samples from the REMC database the mean AUC score is 0.9029. The model is also consistent having a standard deviation of 0.0297 for the AUC score and an AUC score of 0.8313 for the worst performing sample.

The superior performance of PatternChrome can generally be attributed to the extensive feature extraction process. During feature extraction, the algorithm is trained until the training set achieves an AUC score of 99.9%. Therefore, while the algorithm intentionally overfits during the feature extraction phase, the subsequent validation steps (BE and XGBoost hyperparameter tuning) performed on the validation set, mitigate the overfitting. Furthermore, the fact that extracted features and trained models are transferable between samples underline PatternChrome’s generalizability. The approach of searching for spatially invariant patterns in the histone signal data is new for predicting gene expression and the success of PatternChrome underlines the suggestion that there are indeed spatial histone signal patterns that cannot only be used to predict enhancer regions as in the study by Sethi *et al.* (2020) but also to predict transcription levels of individual genes.

Another benefit of PatternChrome is its explainability, as it allows for thorough analysis and visualization of its predictions and the underlying feature contributions. This allows PatternChrome to also be used as a research tool for elucidating gene regulatory mechanisms. This study showed that the patterns predictive of gene expression are mainly found up to 70 bins (3500 bp) upstream of the TSS which is the location where the gene promoter is located for most of the genes. Similarly, the study demonstrated that H3K4me3 is the most influential of the five selected histone modifications for predicting gene expression and that H3K4me3 has a net positive effect on gene expression. These findings are in line with studies that showed that the gene promoter is a vital location for gene regulation as this is the location where transcription initiation occurs and that the histone modification H3K4me3 positively correlates with gene expression Haberle and Stark (2018). H3K36me3 was shown to be the second most gene expression activating histone modification. Interestingly, H3K36me3 exhibited its effect on gene expression predominantly upstream of the TSS which may be surprising considering that studies showed that H3K36me3 is enriched downstream of the TSS Kimura (2013).

Furthermore, using the xgboostExplainer package it is also possible to break down feature contributions on individual genes and study gene regulatory mechanisms at a gene level. Thereby, PatternChrome can identify and visualize histone modification patterns and epigenetic regions which are essential for the regulation of gene expression of individual genes.

While PatternChrome offers excellent performance and can be applied in various ways to explore gene regulatory mechanisms, it does have some limitations.

A limitation of this study is that 1374 (6.94%) genes could not be annotated to a genomic location and were excluded. There might be the possibility that those genes were less predictable and that the performance was better due to leaving out these genes (see Section S9.6, available as supplementary data at *Bioinformatics* online for a brief investigation of RNA-seq distributions of included and excluded genes). Finally, the explainability of the PatternChrome model did not fully address all questions. For example, it is not clear what the learned patterns may point out. It is also not possible to infer any causality from the model or remove conditional dependence from different features.

There are two major ways to build on the research of this study. First, this methodology has the advantage of being out-of-the-box explainable which is not the case for deep learning models, since it has a built-in mechanism to assign scores to the features that contributed positively or negatively to a single classification decision. The scored contributions, in turn, allow to locate spatial patterns around the TSS in ChIP-seq data. Therefore, PatternChrome could be used to further elucidate epigenetic mechanisms. Second, the introduced methodology of finding spatial patterns by using an optimization algorithm coupled to a machine learning model may be of use in other applications such as predicting arrhythmias from electrocardiogram data or forecasting Glucose levels.

While this study showed that there are certain cell-independent patterns regulating gene transcription it is not clear yet what these patterns may represent, how these patterns are interlinked and what the causal direction of these patterns are. Future research could help to understand which histone modifications are causative for gene expression and take up a pioneering role and which histone modifications are more a consequence of gene transcription or are recruited after another histone modification has been established. Transcription factor binding sites (TFBS) may be the missing puzzle piece to understand gene regulation and determine the regions that are important for gene expression. It has been shown that TFBS are interlinked with histone modifications and gene expression Benveniste *et al.* (2014). Similarly, it may be interesting to investigate how the extracted epigenetic patterns differ between healthy and diseased samples and which regions, histone modifications and patterns are associated with these conditions. Pointing out the epigenetic regions that are most important for gene expression may also be useful in the context of pharmacology, personalized medicine or genome engineering as it allows for precise marking of important genetic or epigenetic regions. PatternChrome opens up numerous promising applications in a wide range of scientific and clinical contexts and fields.

In summary, the PatternChrome algorithm relies on an extensive feature extraction process in which PSO is used to select the most predictive spatial features for gene expression level prediction. PatternChrome showed a superior performance for the binary classification of gene expression level based on histone modification features. The algorithm allows for the spatial explanation of its predictions allowing for extensive insights into gene regulatory mechanisms. This approach may offer great opportunities in the fields of both explainable AI and epigenetics.

## Supplementary Material

btaf033_Supplementary_Data

## Data Availability

All required data and the corresponding scripts used to process them are available in an online repository accessible via the link https://gitlab.gwdg.de/MedBioinf/generegulation/patternchrome.
