# Supplementary material for: Prediction of gene expression using histone modification patterns extracted by Particle Swarm Optimization
Source: Bioinformatics. 2025 Jan 29;41(2):btaf033. doi: 10.1093/bioinformatics/btaf033 (PMC11802466; doi:10.1093/bioinformatics/btaf033)
Supplement: btaf033_Supplementary_Data [file btaf033_supplementary_data.zip › 16342_Supplementary_data.pdf]

Table 3. Feature extraction parameters optimised by PSO.

Parameter	Function	Lower bound	Upper bound
Histone modification	Histone modification in whose histone signal the pattern is searched	1*	5*
Start and end position	Start and end bins in the histone signal data where pattern is searched	1	200
Pearson correlation coefficient threshold	Threshold determining from which ρ value onward the pattern and the histone signal at a given location count as a match	0.25	0.75
Pattern width	Width of the constructed pattern	3	7
Pattern anchor points	Points that define the shape of the constructed pattern	0	1

* Number encodes for categorical variable. Each of the five histone modifications is represented by a number: 1 = H3K4me3, 2 = H3K4me1, 3 = H3K36me3, 4 = H3K27me3, 5 = H3K9me3.

Supplementary Data

PSO implementation details

A pattern is represented by a vector of floats between 0 and 1 where the length of the vector corresponds to the pattern length. Each of these numbers serves as an anchor point for a pattern and each anchor point is optimized resulting in an overall pattern shape. The shape of the pattern is correlated with the histone signal arrangement of a selected histone modification in a selected region with equal width. The selected region is determined by two numbers representing the first and last investigated bins. The algorithm imposes a minimal and maximal length of this region. The region must have a length between 21 and 66 bins. These restrictions were made to speed up the optimization process. PSO also selects one of the five histone modification signals for every given pattern. For each bin in the selected region of a selected histone modification the Pearson correlation coefficient (r) is calculated from the pattern and the histone signals in the region. r can be anywhere between -1 and 1 where a positive r indicates that the two compared sets of points have a positive correlation. PSO also optimizes for the r threshold. When this threshold is surpassed at a given bin, this bin is counted as a match. Table 3 shows the parameters and the parameter bounds for PSO.

Within each PSO iteration, PSO calls the objective function 20 times and stores the best fitness value of the iteration. The PSO algorithm uses a constant value of 2.05 for both the cognitive and social coefficient. The inertia weight of PSO starts with a value of 0.8 and linearly decreases to 0.4 up to the maximal number of iterations. PSO continues until the fitness has stagnated for three iterations (stagnation tolerance) or a maximum of 20 iterations is reached. When one PSO round does not result in an improvement of the AUC score the pattern is not stored. PSO then also increases the maximal number of iterations, the swarm size and the stagnation tolerance by one each. When the AUC score improves again, the maximal number of iterations, the swarm size and the stagnation tolerance are reset to the initial settings. Feature extraction continues until the AUC score on the training set is more than or equal to 99.9% or the AUC score did not improve for four PSO rounds.

Alternative prediction models

One drawback of using binary classification as prediction method for gene expression may be that the cutoff between a high and a low gene expression level is suboptimal. In this study the median of the gene expression reads of all genes for a specific sample was chosen in line with the approach of Singh et al. Singh et al. (2016). However, the median is usually not a good cutoff when a distribution is skewed as it is the case in the distribution of gene expression in all samples. Figure 8 shows the distribution of log transformed (via the natural logarithm) gene expression reads of all genes for sample E003. A pseudocount with a value of 0.1 is added to the reads to avoid a log-transformed value of $-\infty$ with reads with a value of 0. As can be seen in Figure 8 the distribution is marked by many genes that are not expressed at all. In addition, for this sample a gaussian-like distribution is present towards the right end of the histogram. The median is marked in Figure 8 and is located at the left side of this gaussian-like distribution. When looking at genes around the median in Figure 8 there is no abrupt change in gene expression level. An examination of the distribution of expression values (figure 8) suggests that genes located near the left side of the median share more epigenetic features with genes on the right side of the median than with non-expressed genes. In addition to the results regarding the binary classification using the median as cutoff, this study presents two alternative approaches that aim to bypass the shortcomings of choosing the median as a binary classification cutoff.

Calculation of realtive feature importance values

The xgboostExplainer package calculates the logit for the binary classification task for each node of a classification model in a specific decision d in a specific sample s. Since each node checks a condition for one feature f (here pattern frequency) these logit values can be attributed to a specific pattern. As depicted in figure 6 logit values are additive, which allows to calculate the ratio of a features logit value I_{fds} and the sum of them in a specific decision. The result of this procedure is a relative importance value for each feature in each decision Q_{fs}. By considering all classification decisions the relative importance of a feature across all decisions can be calculated.

$$Q_{fs} = \frac{\sum_d I_{fds}}{\sum_f \sum_d I_{fds}}$$
$$\sum_f Q_{fs} = 1 \tag{1}$$

Since each feature is searched for in a optimized subregion of the region around the TSS it is possible to map the feature's relative importance to a location relative to the TSS. By adding up the importance values for all features, overlapping a specific bin (50 bp) and dividing it by the overall sum of importance values we can calculate the importance of each 50 bp bin relative to the importance of the other bins.

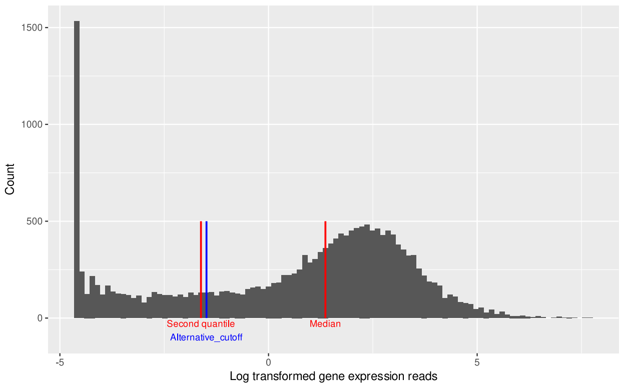

Fig. 8. Histogram of log transformed gene expression reads in sample E003 and demonstration of the alternative gene expression classification cutoff.

Table 4. Comparison of classification metrics between PatternChrome with median cutoff and PatternChrome with the alternative cutoff (reported as mean ± SD). For a metric, the greatest mean value among the two approaches is highlighted bold.

Metric	Median cutoff	Alternative cutoff
AUC	0.9029 ± 0.0297	**0.9269** ± 0.0232
F1 score	**0.8326** ± 0.0330	0.7558 ± 0.0599
Sensitivity	**0.8181** ± 0.0324	0.7612 ± 0.0701
Specificity	0.8527 ± 0.0356	**0.9010** ± 0.0141
Accuracy	0.8354 ± 0.0327	**0.8629** ± 0.0232

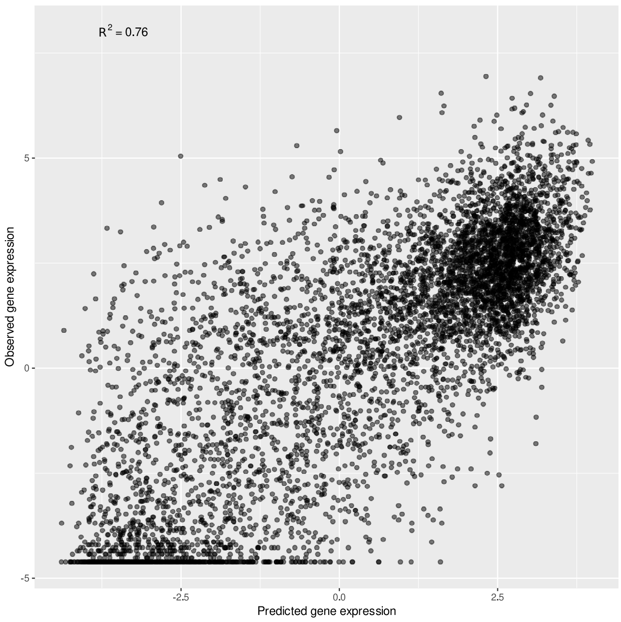

Fig. 9. Scatter plot displaying the predicted gene expression values against the observed gene expression values for sample E003.

Alternative gene expression classification cutoff

As argued by Frasca et al. Frasca et al. (2022) setting the cutoff within the valley between the peak of the gaussian-like distribution and the peak of genes with no expression might be more suitable for binary classification of gene expression. Following their idea, this study proposes an alternative cutoff which is located at the valley between these two peaks. For this, 100 intervals are created ranging from the second quantile to the median. The upper boundary of the interval with the lowest count serves as cutoff.

Table 4 shows the performance for different classification metrics for the PatternChrome algorithm with the median cutoff and the alternative cutoff for metrics in individual samples). The AUC score is the best performing metric in both runs which is unsurprising as PatternChrome optimizes for the best AUC score. The alternative cutoff leads to a better AUC score (gap: 0.024), specificity (gap: 0.0483) and accuracy (gap: 0.0275). On the other side, the median cutoff leads to a greater F1 score (gap: 0.0768) and sensitivity (gap: 0.0569). These differences may partially be a result of the difference in group size: while the groups (i.e. genes with a high and genes with a low gene expression level) are equal when running the algorithm with a median cutoff, the alternative cutoff leads to a greater proportion of genes with a high gene expression level and a lower proportion of genes with a low gene expression level.

Regression

Next to predicting gene expression via grouping genes into categories with either a high or a low gene expression level, one can also directly try to predict the quantity of gene expression using regression. This offers the advantage that no cutoff has to be determined as in a classification task. Before performing regression, the RNA-seq data were modified as described in the previous section via adding a pseudocount and log-transformation. This ensures that the RNA-seq data are more evenly distributed. As XGBoost is also able to work with regression the only parameter that needs to be changed during the PatternChrome workflow is setting the objective of the XGBoost model to "mean squared error regression". Feature extraction was performed again as mentioned in the methods section. The only difference is that during feature extraction the algorithm does not aim to increase the AUC score but to decrease the mean squared error between the predicted results and the observed results. Backward elimination and XGBoost hyperparameter tuning was performed.

Over all 56 samples the mean Pearson correlation coefficient (ρ) is 0.7963 ± 0.0511 and the average mean squared error (MSE) is 2.9621 ± 0.4958. Note that the MSE is dependent on the preprocessing steps and it may also be inappropriate to compare MSEs between different samples due to read quantity differences. ρ may be a more suitable metric to compare the performance of regression between samples as the metric is irrespective of inter sample differences in the read count. Sample E054 achieves the best correlation ($\rho = 0.8662$) while sample E094 has the worst correlation ($\rho = 0.6831$) between all the samples (see section Regression for metrics in individual samples).

Figure 9 plots the predicted expression values against the observed values for the test set of sample E003. Interesting to note here is that PatternChrome is not good in quantifying genes with a gene expression read count of 0 (as can be seen by a wide spread of predicted gene expression for genes with an observed gene expression of around -5).

Investigation of differences in performance between samples

Based on prior results it is clear that some samples generally are harder to predict than others. In this section, possible reasons for these differences and consequences thereof for the PatternChrome algorithm are investigated.

Modeling PatternChrome performance using gene expression and epigenetic sample characteristics

One possible reason that may affect the predictability of a sample is the difference in gene expression distribution. A sample grouped into two groups of genes according to the median cutoff in gene expression reads where the genes of each group have a similarly high gene expression read count within the group but differ in that regard from genes of the other group may be easier to predict. Similarly, a sample with a lot of genes that are not expressed at all may be easier to predict than samples with many genes with an average gene expression.

Another factor for the difference in performance between samples may be differences in the epigenome of the sample. Again, samples where genes within the same group of gene expression level exhibit a more homogeneous epigenetic profile but differ considerably from genes in the other group may be easier to predict.

To investigate these hypotheses a multiple linear regression (MLR) model was created with the aim to predict the mean AUC score per sample from 25 parameters describing gene expression and epigenetic differences. These parameters are sum of reads, standard deviation of reads, difference in sum of reads between genes with high and genes with low gene expression level, difference in standard deviation of reads between genes with and and genes with low gene expression level for RNA-seq, H3K4me3, H3K4me1, H3K36me3, H3K27me3 and H3K9me3 input data as well as number of genes with 0 RNA-seq reads. For RNA-seq, data were log-transformed as described previously. After determining an initial multiple linear regression model from all the parameters, backward elimination was performed where for every run the parameter with the least significant two-tailed p-value was removed from the model until the adjusted R-squared did not further improve.

After backward elimination, 15 variables remained in the MLR model which had an adjusted R squared value of 0.9368. Table 5 displays the five parameters that are most significant in the MLR model.

Of general interest is the parameter estimate which indicates whether the correlation between the parameter and the PatternChrome AUC score is positive or negative. For example, according to the MLR model, a sample with generally more RNA-seq reads tends to have worse performance than a sample with less RNA-seq reads. Concerning histone modifications, H3K36me3 has the greatest influence in the predictability of a sample. The higher the difference in the sum of H3K36me3 reads between the two gene groups the better is the ability of PatternChrome to predict genes in the particular sample. This result indicates that H3K36me3 is the most important histone modification (of the five investigated histone modifications) in stabilizing gene regulation.

Also note that three of the five most important parameters are characterizing the group heterogeneity. These parameters are dependent on the cutoff that splits the genes into two groups. Choosing another cutoff or removing the cutoff altogether (as in regression) may improve the predictive power of the PatternChrome algorithm.

Table 5. The five most significant parameters in the multiple linear regression model to predict AUC scores.

Parameter	Estimate	SE*	p-value
Sum of H3K36me3 reads	$-2.6 * 10^{-08}$	$4.4 * 10^{-09}$	$5.0 * 10^{-07}$
Sum of RNA-seq reads	$-5.9 * 10^{-06}$	$1.0 * 10^{-06}$	$7.6 * 10^{-07}$
Difference in sum of H3K36me3 reads between groups	$4.6 * 10^{-08}$	$8.9 * 10^{-09}$	$7.4 * 10^{-06}$
Difference in sd** of RNA-seq reads between groups	$-4.7 * 10^{-02}$	$9.3 * 10^{-03}$	$1.1 * 10^{-05}$
Difference in sum of H3K4me1 reads between groups	$6.2 * 10^{-08}$	$1.4 * 10^{-08}$	$5.2 * 10^{-05}$

* Standard error.

** Standard deviation.

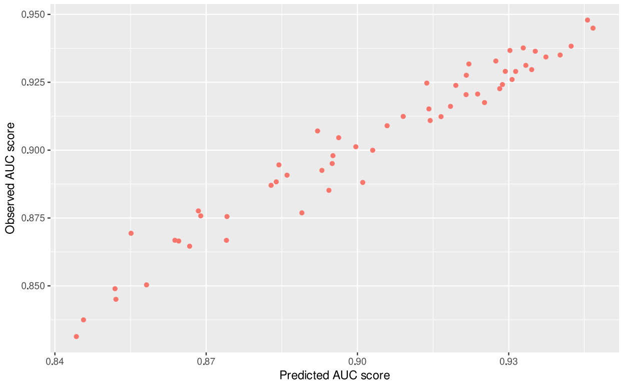

Fig. 10. Scatter plot projecting the observed AUC score against the predicted AUC score according to the previously described MLR model.

Figure 10 plots the predicted AUC scores as determined by the described MLR model against the observed AUC scores for all 56 samples. The Pearson correlation coefficient is 0.9761. This correlation again indicates that the created MLR model is very good in predicting how well the PatternChrome algorithm performs in a particular sample.

Tissue type

The previous section described how differences in RNA-seq and ChIP-Seq profiles between samples are connected to the differences in performance between sample. The tissue type may conversely be responsible for the differences in the RNA-seq and ChIP-Seq profiles. Figure 11 plots the distribution of the AUC score of all 56 samples grouped by tissue type (see section Data preprocessing for the description of samples and their tissue type). amples of tissue types sample cell line (CL), primary culture (PCU) and primary cell (PC) exhibit on average the greatest AUC score (AUC $= 0.9305 \pm 0.0113$, 0.9253 ± 0.0156 and 0.9223 ± 0.0080, respectively). On the other hand, samples of the category embryonic stem cell (ESC), embryonic stem cell-derived (ESCD) and particularly primary tissue (PT) perform considerably worse (AUC $= 0.9043 \pm 0.0167$, $0.8971 \pm$

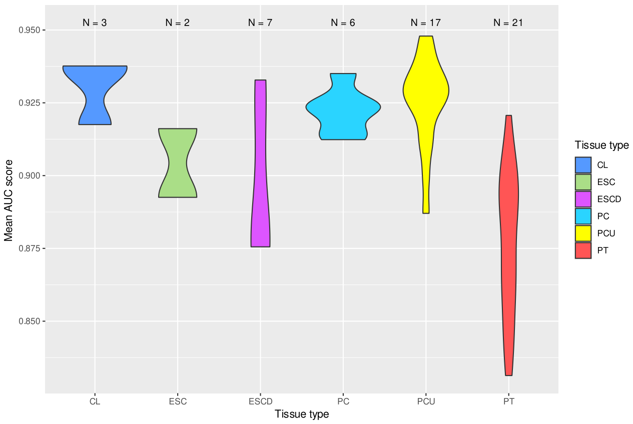

Fig. 11. Distribution of mean AUC scores for samples grouped by tissue type (Abbreviations: CL = Cell line, ESC = Embryonic stem cell, ESCD = Embryonic stem cell-derived, PC = Primary cell, PCU = Primary culture, PT = Primary tissue).

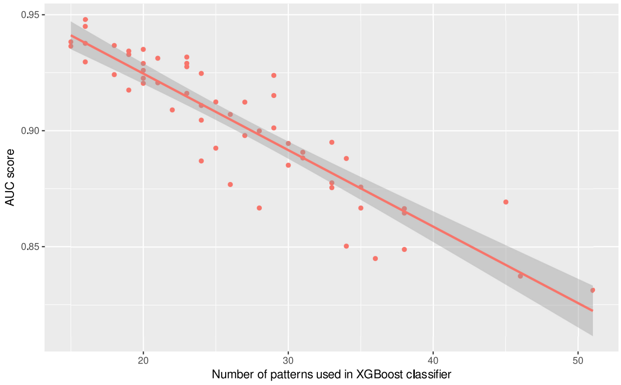

Fig. 12. Correlation between number of extracted patterns and AUC score over all 56 samples.

0.0268 and 0.8772 ± 0.0249, respectively). The findings show that tissue type correlates well with the performance of the PatternChrome algorithms. Potential reasons for this may be the tissue heterogeneity, as samples of tissue type PT may harbour more than one biological cell type while this is usually not the case for cultured tissue types such as CL,PCU,PC,ESC and ESCD.

Correlation of pattern number and classification performance

After performing feature extraction and backward elimination the number of patterns (features) may differ in different samples. The correlation of the number of patterns used in the final XGBoost model and the mean AUC score for each sample is shown in Figure 12. The figure indicates a strong negative correlation between the two variables (Pearson correlation coefficent = -0.9049). Here it is important to note that no causation can be inferred from this correlation. Although it may be possible that a complex model with more features (i.e. patterns) tends to overfit and thereby perform suboptimal, it may also be possible that a sample whose gene expression is inherently less predictable may require more patterns that are less generic and fail to fully map the gene regulatory mechanisms of the sample.

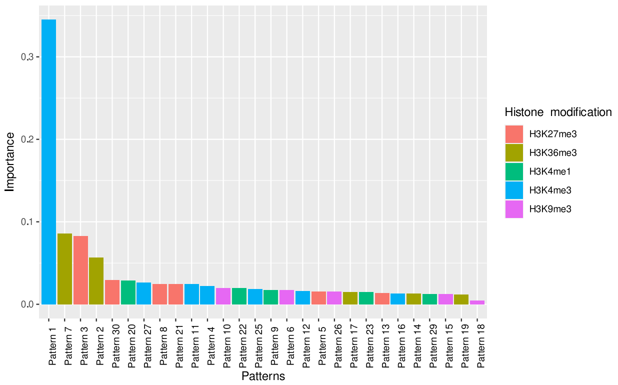

Fig. 13. Feature importance of different patterns for the XGBoost classification model for E003.

Explainability at sample level

The PatternChrome classifier is trained on each sample separately. Thus, to demonstrate the decision making process of the classifier, the classification process for the sample E003 (H1 embryonic stem sample) is analysed in detail in this section.

Feature importance of individual patterns

Figure 13 shows the mean feature importance of individual trained patterns to the XGBoost classifier for sample E003. In this sample 28 patterns were learned in the feature extraction process. Pattern 1 (the number indicates the order in which the pattern was added to the feature matrix during feature extraction), had the greatest importance to the XGBoost model (34.49%). Two other patterns, Pattern 7 and Pattern 3, contributed each around 8%. Other patterns contributed relatively little with 17 patterns contributing less than 2% to the XGBoost model.

Correlation of pattern frequency and pattern contribution

Next to the importance of the learned patterns, the correlation coefficient (ρ) of pattern frequency and the net pattern contributions to the XGBoost model can give a hint of whether the pattern may have an activating or an inhibiting effect on gene expression. The feature most important for sample E003, pattern 1, has a correlation of 0.9545. 14 of the 28 patterns exhibit a negative correlation and 14 a positive one. 7 patterns show a generally weak absolute (i.e. positive or negative) correlation ranging from -0.5 to 0.5 see Figure 14.

Visualising the association between pattern frequency and pattern correlation for the two patterns with the highest feature importance

A drawback of using ρ to indicate whether a pattern acts positively or negatively and assess the strength of the correlation is that ρ assumes a linear correlation between pattern frequency and the contributions. To investigate the nature of the correlation the pattern frequency and the prediction contributions of the two patterns with the highest feature importance, pattern 1 and pattern 7 were plotted in Figure 15. The fitted lines for both pattern 1 and pattern 7, which is based on a generalized additive model (GAM) assuming non-linear relations, is sigmoidal-shaped. A steep increase in the prediction contribution of Pattern 1 is seen when the pattern frequency is around 3 to 7. For Pattern 7 the steep

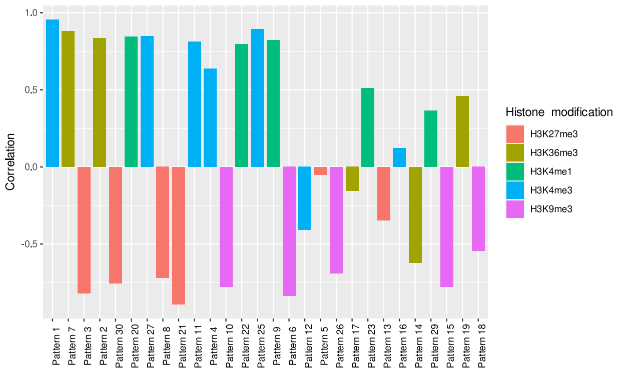

Fig. 14. Correlations of pattern contribution and pattern frequency for sample E003.

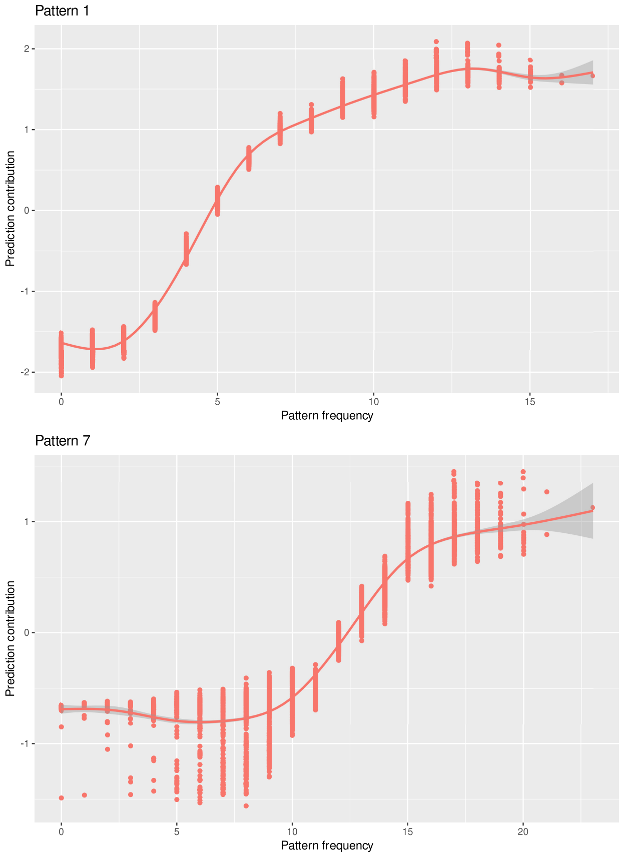

Fig. 15. Relation between pattern frequency and the prediction contributions for Pattern 1 and Pattern 7 in sample E003.

increase occurs around a pattern frequency between 10 to 15. Pattern 1 exhibits a lower spread around the fitted line than Pattern 7. This indicates that the contribution of Pattern 1 is more independent from other patterns while for Pattern 7 the contribution depends considerably on the pattern frequencies of other patterns.

Decision boundary visualization

To demonstrate how XGBoost uses the pattern frequencies to predict gene expression for an individual sample the decision

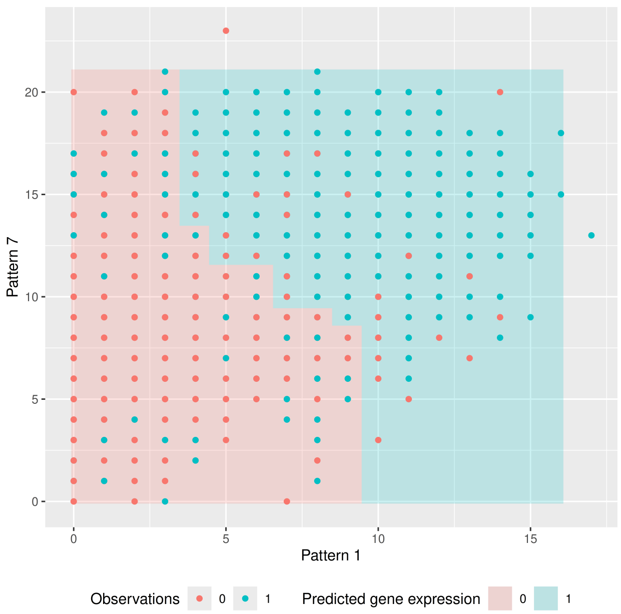

Fig. 16. Decision boundary of a simplified XGBoost model based on the two most contributing patterns for sample E003. Note that not all observations of the test set are displayed. When two or more observations in the test set have the same feature combination, only the last observed one is shown

boundary was plotted for a simplified XGBoost model based on the two most contributing features, pattern 1 and pattern 7 (see Figure 16). For the decision boundary, XGBoost was trained on the training set with only the two selected features and predictions were made for each possible combination of the two features. The observations of the test set were added to the decision boundary to give an impression of how well the decision boundary fits (notice that not all observations of the test set are displayed. When two or more observations in the test set have the same feature combination, only the last observed one is shown). The created decision boundary is non-linear. The decision boundary is dominated by pattern 1 while pattern 7 is only of importance when genes have a frequency of pattern 1 between around 2 to 9. This observation is in line with the relation between pattern frequency and prediction contribution (see Figure 15) and the feature importance (see Figure 13) described earlier.

Visualization of pattern matches in histone signal data

To demonstrate which regions are marked by the trained patterns, pattern 1 was selected for demonstration purpose as it has the highest importance for sample E003. Pattern 1 is to be found in bins -40 to -12 of the binned H3K4me3 ChIP-seq data and has an optimized ρ threshold of 0.6124. Pattern 1 has a width of 5 and is characterized by a peak followed by an increase and a subsequent plateau in the ChIP-seq signal (see Figure 17).

To better understand which specific epigenome regions match with this pattern, the selected epigenomic regions for pattern 1 (bins -40 to -12) of the genes OCT4 and TIE1, along with an associated graph displaying ρ between pattern 1 and the H3K4me3 signal data at each bin are illustrated in figure

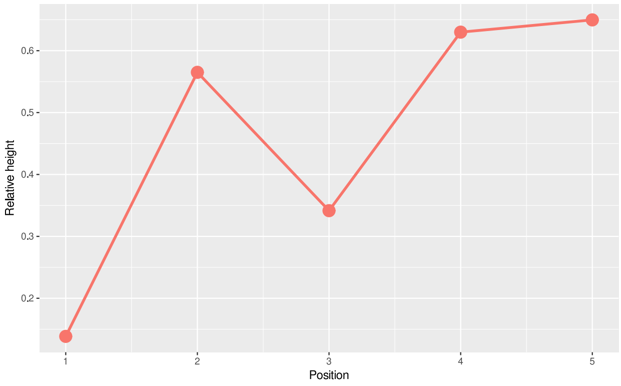

Fig. 17. Shape of Pattern 1 in sample E003.

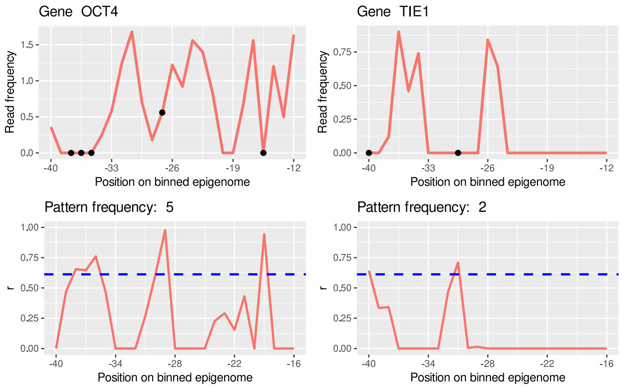

Fig. 18. Visualization of matches between the selected pattern and binned H3K4me3 signal data of genes OCT4 and TIE1.

18. In the two upper graphs of figure 18 the black dots mark the bins at which a match is detected. Both lower graphs also indicate the frequency of pattern matches in the selected region. For these two genes, two different frequencies are observed in the same region (OCT4 pattern frequency = 5, TIE1 pattern frequency = 2). Note that a match is determined by an ρ value higher than the optimized threshold (0.6124), which is generally thought of as a weak correlation.

Comparison of RNA Seq distributions of included and excluded genes

To investigate how genes that were excluded from the study (see section Data preprocessing for why the genes were left out) may have impacted the predictions, the log-transformed RNA-seq read distribution of the 1374 excluded genes in all 56 samples were compared against the one of the 18421 included genes in all 56 samples. The two distributions are shown in Figure 19. Included genes are colored in teal and excluded genes are colored n orange. The mean read value of included genes was 0.8773 $\pm$ 2.2403 while the mean read value of the excluded genes was -0.6763 $\pm$ 1.9707. While from the included genes 11.69 % had 0 RNA-seq reads, 31.08% of excluded genes had 0 RNA-seq reads. When assuming an RNA-seq distribution over all 56 samples with included and excluded genes, 79.36% of excluded genes would be below the median of this distribution while 47.81% of included genes would be below the median. These outcomes suggest that most of the excluded genes had no expression or a low expression level. It is also likely that the excluded genes would have been easier to predict than most included genes as many of the excluded genes were far below the median cutoff point.

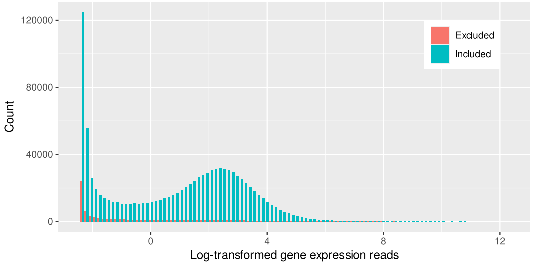

Fig. 19. RNA Seq distribution of included and excluded genes over all 56 samples.

Aggregated and sample-specific classification metrics using the median as cutoff

Table 6. Table of sample specific classification metrics for binary classification using the median as gene expression cutoff

Sample	AUC	F1 score	Sensitivity	Specificity	Accuracy
Mean	0.9029 ± 0.0295	0.8326 ± 0.0327	0.8181 ± 0.0321	0.8527 ± 0.0353	0.8354 ± 0.0324
E003	0.9000	0.8277	0.8231	0.8341	0.8286
E004	0.8755	0.8132	0.8139	0.8121	0.8130
E005	0.8769	0.8125	0.8160	0.8074	0.8117
E006	0.8758	0.8043	0.7801	0.8403	0.8102
E007	0.8776	0.8084	0.7957	0.8271	0.8114
E011	0.9090	0.8391	0.8315	0.8494	0.8405
E012	0.9328	0.8673	0.8525	0.8866	0.8696
E013	0.9317	0.8553	0.8288	0.8907	0.8598
E016	0.9161	0.8350	0.8190	0.8572	0.8381
E024	0.8925	0.8177	0.8075	0.8325	0.8200
E027	0.9238	0.8448	0.8251	0.8717	0.8484
E028	0.9109	0.8469	0.8309	0.8687	0.8498
E037	0.9152	0.8473	0.8298	0.8711	0.8504
E038	0.9247	0.8593	0.8593	0.8592	0.8592
E047	0.9226	0.8602	0.8434	0.8826	0.8630
E050	0.9350	0.8721	0.8583	0.8900	0.8741
E053	0.9204	0.8525	0.8244	0.8904	0.8574
E054	0.9276	0.8590	0.8369	0.8883	0.8626
E055	0.9367	0.8685	0.8505	0.8920	0.8713
E056	0.9124	0.8363	0.8143	0.8670	0.8406
E057	0.9242	0.8569	0.8478	0.8690	0.8584
E058	0.9449	0.8811	0.8674	0.8985	0.8829
E059	0.8870	0.8115	0.7899	0.8430	0.8164
E061	0.9383	0.8741	0.8559	0.8975	0.8767
E062	0.9123	0.8410	0.8339	0.8508	0.8423
E065	0.8313	0.7660	0.7486	0.7939	0.7713
E066	0.8980	0.8210	0.8078	0.8399	0.8239
E070	0.9046	0.8326	0.8099	0.8643	0.8371
E071	0.8490	0.7695	0.7517	0.7980	0.7748
E079	0.8646	0.7860	0.7662	0.8166	0.7914
E082	0.8881	0.8109	0.7899	0.8416	0.8158
E084	0.8908	0.8130	0.7957	0.8382	0.8170
E085	0.8951	0.8200	0.8116	0.8321	0.8219
E087	0.8852	0.8111	0.7984	0.8298	0.8141
E094	0.8375	0.7621	0.7517	0.7790	0.7654
E095	0.9013	0.8272	0.8112	0.8497	0.8305
E096	0.8694	0.7935	0.7892	0.8000	0.7946
E097	0.9071	0.8353	0.8207	0.8555	0.8381
E098	0.8451	0.7599	0.7493	0.7770	0.7632
E100	0.9207	0.8451	0.8427	0.8484	0.8455
E104	0.8883	0.8100	0.7977	0.8281	0.8129
E105	0.8946	0.8197	0.8058	0.8396	0.8227
E106	0.8504	0.7766	0.7581	0.8058	0.7819
E109	0.8668	0.7961	0.7794	0.8213	0.8004
E112	0.8668	0.8036	0.8082	0.7966	0.8024
E113	0.8665	0.7982	0.7994	0.7963	0.7978
E114	0.9175	0.8497	0.8204	0.8893	0.8548
E116	0.9343	0.8775	0.8674	0.8904	0.8789
E117	0.9364	0.8748	0.8603	0.8934	0.8768
E118	0.9376	0.8752	0.8552	0.9008	0.8780
E119	0.9290	0.8635	0.8461	0.8863	0.8662
E120	0.9260	0.8591	0.8332	0.8934	0.8633
E122	0.9290	0.8598	0.8440	0.8805	0.8623
E123	0.9479	0.8849	0.8789	0.8924	0.8856
E127	0.9312	0.8637	0.8349	0.9015	0.8682
E128	0.9297	0.8671	0.8468	0.8937	0.8702
