# Supplementary figures and images for: Prediction of gene expression using histone modification patterns extracted by Particle Swarm Optimization
Source: Bioinformatics. 2025 Jan 29;41(2):btaf033. doi: 10.1093/bioinformatics/btaf033 (PMC11802466; doi:10.1093/bioinformatics/btaf033)

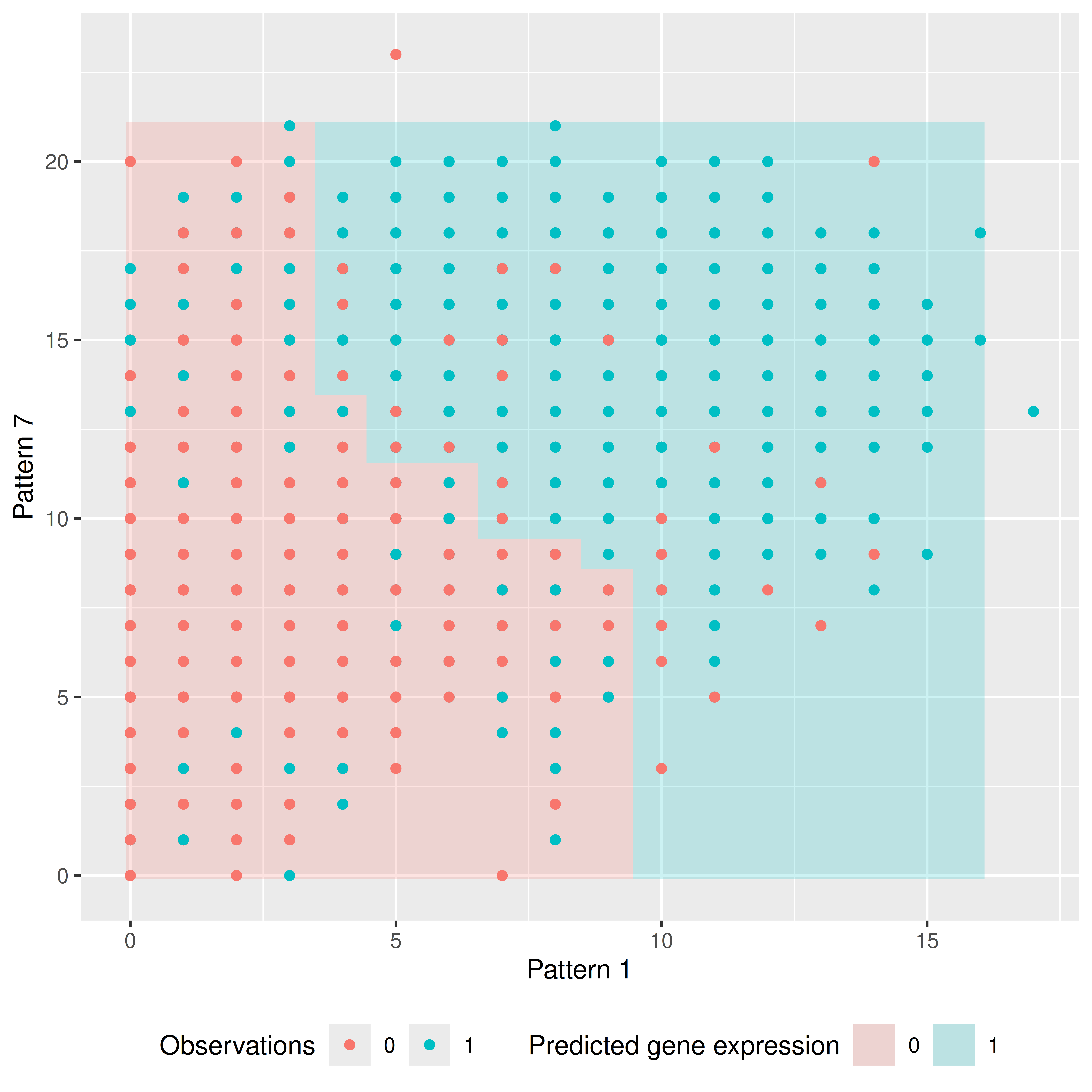

Supplement: btaf033_Supplementary_Data [file btaf033_supplementary_data.zip › d6b00_Figure_16_SI9.png]
